# Hospital Use and Mortality Among Decarcerated Individuals With Substance Use Disorder After a Large-scale COVID-19 Emergency Prison Release Program

**DOI:** 10.1001/jamahealthforum.2023.1200

**Published:** 2023-06-02

**Authors:** Peter Treitler, Molly Nowels, Kenneth A. Feder, Brendan Saloner, Rusty Reeves, Lisa DeBilio, Stephen Crystal

**Affiliations:** 1 Institute for Health, Health Care Policy and Aging Research, Rutgers University, New Brunswick, New Jersey; 2 School of Social Work, Rutgers University, New Brunswick, New Jersey; 3 Department of Health Behavior, Society, and Policy, Rutgers School of Public Health, Piscataway, New Jersey; 4 Department of Mental Health, Johns Hopkins Bloomberg School of Public Health, Baltimore, Maryland; 5 Department of Health Policy and Management, Johns Hopkins Bloomberg School of Public Health, Baltimore, Maryland; 6 Rutgers University Correctional Health Care, Trenton, New Jersey; 7 Department of Psychiatry, Robert Wood Johnson Medical School, Rutgers University, Piscataway, New Jersey

## Abstract

This cohort study examines hospital use and mortality among persons with substance use disorder (SUD) who were released from New Jersey state prisons after a COVID-19 emergency prison release program.

## Introduction

New Jersey enacted the Public Health Emergency Credit Act (PHECA) to reduce virus transmission among incarcerated persons, shortening sentences by up to 8 months for residents of state prisons during the COVID-19 public health emergency (PHE).^1^ As a result, New Jersey experienced the largest decrease in its prison population among all states.^2,3^ There were concerns that this mass release would overwhelm reentry services,^4^ especially for individuals with substance use disorders (SUDs), who have a high risk of postrelease overdose and acute care use.^5^ We examined whether these risks changed during the PHECA period compared with the pre-PHECA period.

## Methods

We analyzed releases among incarcerated individuals with SUDs released from New Jersey state prisons from 2019 to 2020. For each release, we linked state Department of Corrections records to state Department of Health all-payer hospital discharge and mortality records. Using Cox proportional hazards models, we estimated hazard ratios (HRs) for the 45-day postrelease occurrence of SUD-related acute care visits (ACVs; emergency department or inpatient visits), overdose-related ACVs, and death from overdose. Events in 3 periods were compared : period 1, before the COVID-19 PHE (January 1, 2019, to March 10, 2020); period 2, during the PHE but before PHECA implementation (March 11 to November 3, 2020); and period 3, after PHECA implementation (November 4 to December 31, 2020). Models were adjusted for demographic characteristics (age, sex, self-reported race and ethnicity, education, and marital status) and incarceration characteristics (index offense type, index offense severity, parole release, and participation in each of 11 prerelease programs). Postrelease outcomes were censored at 45 days or the end of the study (eMethods in Supplement 1). Analyses were conducted from July to September 2022 using SAS Enterprise Guide 8.3 (SAS Institute Inc). A 2-sided *P* value of <.05 was considered statistically significant. This cohort study was approved by the Rutgers University Institutional Review Board, which waived the informed consent requirement because we used secondary data collected for nonresearch purposes. We followed the STROBE reporting guideline.

## Results

Of 11 177 releases among 10 115 incarcerated individuals (median age at release, 34 [IQR, 28-43] years), 749 (6.7%) were females and 10 428 (93.3%) were males. Releases during period 1 comprised 57.0% of releases; 25.6% occurred during period 2 and 17.4% occurred during period 3. Cohorts were similar across periods except that individuals released during period 3 were less likely to be on parole (10.0% vs 44.2% and 47.3% for periods 1 and 2, respectively; Table). During the first 45 days after release, an SUD-related ACV occurred in 526 releases (4.7%) and death from overdose occurred in 32 releases (0.3%). In adjusted models, the risk for SUD-related ACVs did not differ between the 3 periods. Adjusted HRs for any nonfatal overdose-related ACV and fatal overdose were lower for individuals released in period 3 compared with before the PHE, but the differences were not statistically significant (Figure).

**Table.** Selected Characteristics of Releases of Individuals With Substance Use Disorder From New Jersey Prisons From 2019 to 2020

Characteristic	Releases, No. (%) (N = 11 177)			*P* value^b^
	Period 1^a^	Period 2^a^	Period 3^a^	
No. of releases	6374 (57.0)	2860 (25.6)	1943 (17.4)	NA
Outcomes within 45 d of release				
Any SUD-related acute care visit	304 (4.8)	137 (4.8)	85 (4.4)	.75
Any overdose-related acute care visit	81 (1.3)	38 (1.3)	16 (0.8)	.23
Overdose death^c^	19 (0.3)	<11	<11	.96
Demographic characteristics				
Age at release, y				
18-34	3271 (51.3)	1466 (51.3)	1072 (55.2)	.01
35-44	1678 (26.3)	751 (26.3)	508 (26.1)	
45-64	1387 (21.8)	625 (21.9)	349 (18.0)	
≥65	38 (0.6)	18 (0.6)	14 (0.7)	
Sex				
Female	479 (7.5)	170 (5.9)	101 (5.2)	<.001
Male	5896 (92.5)	2690 (94.1)	1842 (94.8)	
Race and ethnicity				
Hispanic	844 (13.3)	402 (14.1)	258 (13.3)	<.003
Non-Hispanic Black	3480 (54.6)	1590 (55.6)	1155 (59.4)	
Non-Hispanic White	1954 (30.7)	826 (28.9)	512 (26.4)	
Non-Hispanic Other^d^	92 (1.4)	41 (1.4)	18 (0.9)	
Unknown	4 (<0.1)	1 (<0.1)	0	
Education level				
<High school	1535 (24.1)	676 (23.6)	501 (25.8)	<.001
High school diploma	4258 (66.8)	1954 (68.3)	1254 (64.5)	
Postsecondary education	396 (6.2)	144 (5.0)	99 (5.1)	
Unknown	185 (2.9)	86 (3.0)	89 (4.6)	
Marital status				
Not married	5723 (89.8)	2583 (90.3)	1762 (90.7)	.55
Married	400 (6.3)	173 (6.1)	103 (5.3)	
Unknown	251 (3.9)	104 (3.6)	78 (4.0)	
Release type				
Parole	2819 (44.2)	1352 (47.3)	195 (10.0)	<.001
Not parole	3536 (55.5)	1508 (52.7)	1748 (90.0)	
Unknown	19 (<0.1)	0	0	

Abbreviations: NA, not applicable; SUD, substance use disorder.

^a^ Period 1, before the COVID-19 pandemic public health emergency, January 1, 2019, to March 10, 2020; period 2, during the public health emergency but before the Public Health Emergency Credit Act, March 11 to November 3, 2020); and period 3, after the Public Health Emergency Credit Act, November 4 to December 31, 2020.

^b^ *P* values determined from χ^2^ tests.

^c^ Cells with fewer than 11 data points are not reported in accordance with data use agreements.

^d^ Includes persons who reported their race as Asian, American Indian or Alaska Native, or other (not defined).

**Figure.**
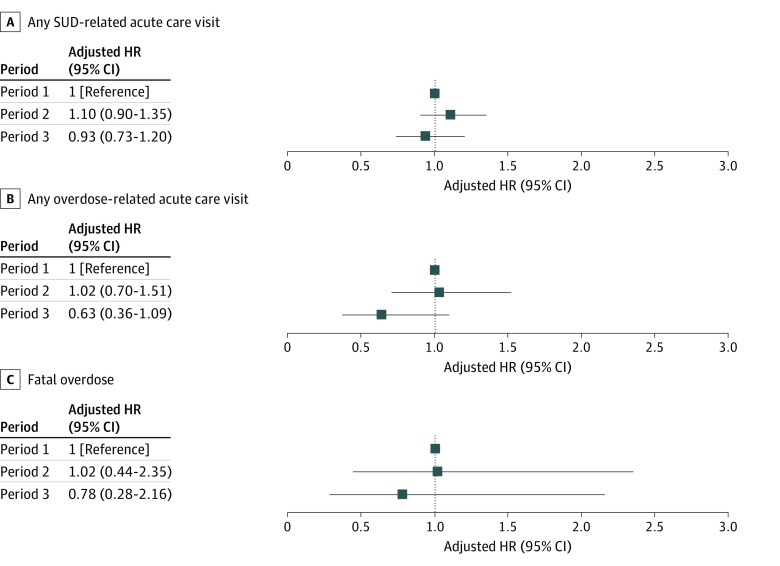
Adjusted Hazard Ratios of Acute Care Utilization and Fatal Overdose During 45 Days After Release From Prison Period 1 was from January 1, 2019, to March 10, 2020 (before the COVID-19 public health emergency [PHE]), period 2 was from March 11 to November 3, 2020 (during the PHE but before implementation of the Public Health Emergency Credit Act [PHECA]), and period 3 was from November 4 to December 31, 2020 (after PHECA implementation). Hazard ratios (HRs) and CIs were calculated using Cox proportional hazards regression models adjusted for age, sex, race and ethnicity, education, marital status, index offense type, index offense severity, parole release, and participation in each of 11 prerelease programs (eMethods in Supplement 1). SUD indicates substance use disorder.

## Discussion

New Jersey’s large-scale decarceration in response to the COVID-19 pandemic was unprecedented. While PHECA strained the state’s reentry system and access to SUD care,^4^ we found no evidence that postrelease rates of SUD-related ACVs or overdose death were higher for individuals with SUD released after the PHECA than during earlier periods. Decarceration efforts during the pandemic were important for mitigating virus transmission.^6^ New Jersey has robust reentry supports for incarcerated individuals with SUD, including Medicaid enrollment, providing prerelease medications for opioid use disorder, and a statewide peer navigator program, all of which may have lowered postrelease risks.^4^ Study limitations include the lack of data on overdoses not resulting in ACVs or death, and data were limited to individuals with SUD. Also, the observational study design may not fully adjust for differences in cohort characteristics over time (eg, insurance status), and results may not be generalizable to other states. Nonetheless, New Jersey’s decarceration experience may provide a model for future policy reform as correctional systems seek to reduce incarceration without increasing health risk.

## Notes

Supplement 1.
eMethods

eReference

Supplement 2.
Data Sharing Statement

## References

[1] Official Site of the State of New Jersey. Governor Murphy signs legislation requiring public health emergency credits to be awarded to certain inmates and parolees during a public health emergency. October 19, 2020. Accessed November 22, 2022. https://www.nj.gov/governor/news/news/562020/20201019c.shtml.

[2] Yi K. NJ’s COVID-19 prison release program restarts Thursday with 260 freed early. *Gothamist*. February 9, 2022. Accessed November 22, 2022. https://gothamist.com/news/njs-covid-19-prison-release-program-restarts-thursday-with-260-freed-early

[3] Carson EA, Nadel M, Gaes G. *Impact of COVID-19 on State and Federal Prisons, March 2020–February 2021.* US Department of Justice, Office of Justice Programs, Bureau of Justice Statistics. Bulletin NCJ 304500. August 2022. Updated December 20, 2022. Accessed January 3, 2023. https://bjs.ojp.gov/content/pub/pdf/icsfp2021.pdf

[4] Bono MH, Treitler P, Saloner B, Crystal S. Returning home during the pandemic: a thematic analysis describing experiences of people with substance use disorders released early from New Jersey prisons during COVID-19. Health Justice. 2023;11(1):11. doi:10.1186/s40352-023-00208-x 36847934 PMC9969013

[5] Mital S, Wolff J, Carroll JJ. The relationship between incarceration history and overdose in North America: a scoping review of the evidence. Drug Alcohol Depend. 2020;213:108088. doi:10.1016/j.drugalcdep.2020.108088 32498032 PMC7683355

[6] Wang EA, Western B, Backes EP, Schuck J, eds; Committee on the Best Practices for Implementing Decarceration as a Strategy to Mitigate the Spread of COVID-19 in Correctional Facilities. Decarcerating Correctional Facilities during COVID-19: Advancing Health, Equity, and Safety. National Academies Press; 2020. 33411438

