# Supplementary material for: Hospital Use and Mortality Among Decarcerated Individuals With Substance Use Disorder After a Large-scale COVID-19 Emergency Prison Release Program
Source: JAMA Health Forum. 2023 Jun 2;4(6):e231200. doi: 10.1001/jamahealthforum.2023.1200 (PMC10238949; doi:10.1001/jamahealthforum.2023.1200)
Supplement: Supplement 1. — eMethods eReference [file jamahealthforum-e231200-s001.pdf]

Supplemental Online Content

Treitler P, Nowels M, Feder KA, et al. Hospital use and mortality among decarcerated individuals with substance use disorder after a large-scale COVID-19 emergency prison release program. *JAMA Health Forum.* 2023;4(6):e231200. doi:10.1001/jamahealthforum.2023.1200

eMethods

eReference

This supplementary material has been provided by the authors to give readers additional information about their work.

© 2023 Treitler P et al. *JAMA Health Forum.*

eMethods

Data Sources and Linkage

This study used linked administrative databases from 3 sources:

1. *New Jersey Department of Corrections (NJDOC) administrative datafiles for releases in 2019-2020:* Contains admission/release dates, inmate demographics, substance use disorder (SUD) diagnoses, service and program participation, parole status, and offense characteristics.

2. *New Jersey Department of Health (NJDOH) uniform billing hospital inpatient and emergency department (ED) discharge data for 2019-2020:* Includes all-payer inpatient/ED health care claims for all ED visits and hospitalizations in the post-release period.

3. *NJDOH mortality records for 2019-2020:* Contains detailed mortality and cause of death data for all deaths occurring in New Jersey.

Datasets were linked in a CMS- and HIPAA-compliant facility with state-of-the-art protections for highly sensitive data, using tested, multi-phase probabilistic matching methods that minimize false positive matches while maximizing correct linkage of records that represent the same individual. Identifiers used in the linkage included first name, last name, middle initial, date of birth, city, zip code, and sex. Following linkage, direct identifiers were removed from the dataset and replaced with a random identifier, and deidentified analytic data files were transferred to a separate computing platform for analysis.

Cohort Definition

The study cohort included 11,177 releases among 10,115 individuals from 2019-2020. We included individuals if they met the following criteria: 1) released from a New Jersey state prison between January 1, 2019 and December 31, 2020; 2) aged 18 or older at release; and 3) had a SUD, as defined by a documented SUD diagnosis or Texas Christian University Drug Screen[1] score of 1 or more.

Measures

Outcomes

Fatal overdose: Overdose deaths within 45 days of release were identified using underlying cause of death ICD-10 codes contained in 2019-2020 NJDOH mortality records. ICD-10 codes included X40-X44, X60-X64, X85, and Y10-Y14.

Any SUD acute care visit: We used 2019-2020 NJDOH all-payer hospital discharge data to identify SUD-related ED and inpatient encounters in the 45 days after release. SUD-related encounters were defined as any visits with ICD-10-CM primary or secondary diagnosis codes for SUD or drug poisoning. ICD-10-CM codes are shown in the eTable.

Overdose-related acute care visit: We used 2019-2020 NJDOH all-payer hospital discharge data to identify ED and inpatient encounters for drug overdose in the 45 days after release. Drug overdose encounters were defined as any visits with ICD-10-CM primary or secondary diagnosis codes for drug poisoning, using poisoning ICD-10-CM codes shown in the eTable.

Predictors/covariates:

The following covariates/predictors are from 2019-2020 NJDOC administrative datafiles:

Time period: The study period was divided into 3 time periods based on individuals' release dates: period 1, before the COVID-19 public health emergency (PHE) (1/1/2019-3/10/2020); period 2, during

© 2023 Treitler P et al. *JAMA Health Forum.*

the PHE but before the early release legislation (3/11/2020-11/3/2020), and period 3, the mass-release period following the early release legislation (11/4/2020-12/31/2020).

Age at release: Calculated by subtracting release date from date of birth, then categorized into 18-34, 35-44, 45-64, and 65+.

Sex: Categories of female and male, as documented in NJDOC administrative datafiles.

Race and ethnicity: Incarcerated persons self-reported their race at intake, using the following categories: American Indian/Alaska Native, Asian, Black, White, or Other. Incarcerated persons separately indicated their ethnicity as Hispanic/Latino or non-Hispanic/Latino. For this study, we collapsed race and ethnicity into the following categories: Black, non-Hispanic; Hispanic; White, non-Hispanic; other, non-Hispanic; and unknown. Individuals of any race who indicated their ethnicity as Hispanic/Latino were categorized as Hispanic. Other, non-Hispanic includes Asian, American Indian/Alaska Native, and Other.

Education level: NJDOC administrative datafiles contained a variable for highest level of education ranging from 1st grade to doctorate degree. These were categorized into less than high school, high school diploma, post-secondary education, and unknown.

Marital status: DOC administrative datafiles contained a variable for marital status. Responses were recoded into married and not married (i.e., single, separated, divorced, or widowed).

Index offense category: Index offense was determined from a variable indicating the most serious offense associated with the incarceration. Using offense descriptions, these were categorized into the following types: drug, property, supervision, violent, weapons, and other.

Index offense severity: NJDOC administrative datafiles contained a numeric indicator of offense severity, which we recoded to 1 (e.g., parole violation) through 6 (e.g., 1st degree violent crimes including homicide).

Parole status at release: Categorized as parole and no parole (for individuals who were released following completion of their full sentences).

Pre-release program participation: NJDOC administrative datafiles contained variables indicating participation (yes/no) in each of the following pre-release programs: Adult Mandatory Education, Vocational/Education Program, Residential Community Release Program, Mid-State Correctional Facility SUD Treatment Program, AA/NA/GA, other in-prison SUD treatment, Living-in-Balance curriculum, Engaging the Family curriculum, Anger Management curriculum, or Work Release. Models included separate variables for each program.

© 2023 Treitler P et al. *JAMA Health Forum.*

eTable. ***ICD-10-CM*** **diagnosis codes for substance use disorders and drug poisoning**

**Opioid use disorder**<br>F11.1, F11.10, F11.12, F11.120, F11.121, F11.122, F11.129, F11.14, F11.15, F11.150, F11.151, F11.159, F11.181, F11.182, F11.188, F11.19, F11.2, F11.20, F11.22, F11.220, F11.221, F11.222, F11.229, F11.23, F11.230, F11.24, F11.25, F11.250, F11.251, F11.259, F11.281, F11.282, F11.288, F11.29
**Other substance use disorders**<br>F10.1, F10.10, F10.12, F10.120, F10.121, F10.129, F10.14, F10.15, F10.150, F10.151, F10.159, F10.18, F10.180, F10.181, F10.182, F10.188, F10.19, F10.2, F10.20, F10.22, F10.220, F10.221, F10.229, F10.23, F10.230, F10.231, F10.232, F10.239, F10.24, F10.240, F10.25, F10.250, F10.251, F10.259, F10.26, F10.27, F10.28, F10.280, F10.281, F10.282, F10.288, F10.29, F12.1, F12.10, F12.12, F12.120, F12.121, F12.122, F12.129, F12.15, F12.150, F12.151, F12.159, F12.18, F12.180, F12.188, F12.19, F12.2, F12.20, F12.22, F12.220, F12.221, F12.222, F12.229, F12.23, F12.230, F12.25, F12.250, F12.251, F12.259, F12.28, F12.280, F12.288, F12.29, F13.1, F13.10, F13.12, F13.120, F13.121, F13.129, F13.14, F13.15, F13.150, F13.151, F13.159, F13.18, F13.180, F13.181, F13.182, F13.188, F13.19, F13.2, F13.20, F13.22, F13.220, F13.221, F13.229, F13.23, F13.230, F13.231, F13.232, F13.239, F13.24, F13.240, F13.25, F13.250, F13.251, F13.259, F13.26, F13.27, F13.28, F13.280, F13.281, F13.282, F13.288, F13.29, F14.1, F14.10, F14.12, F14.120, F14.121, F14.122, F14.129, F14.14, F14.15, F14.150,F14.151, F14.159, F14.18, F14.180, F14.181, F14.182, F14.188, F14.19, F14.2, F14.20, F14.22, F14.220, F14.221, F14.222, F14.229, F14.23, F14.230, F14.24, F14.240, F14.25, F14.250, F14.251, F14.259, F14.28, F14.280, F14.281, F14.282, F14.288, F14.29, F15.1, F15.10, F15.12, F15.120, F15.121, F15.122, F15.129, F15.14, F15.15, F15.150, F15.151, F15.159, F15.18, F15.180, F15.181, F15.182, F15.188, F15.19, F15.2, F15.20, F15.22, F15.220, F15.221, F15.222, F15.229, F15.23, F15.230, F15.24, F15.240, F15.25, F15.250, F15.251, F15.259, F15.28, F15.280, F15.281, F15.282, F15.288, F15.29, F16.1, F16.10, F16.12, F16.120, F16.121, F16.122, F16.129, F16.14, F16.15, F16.150, F16.151, F16.159, F16.18, F16.180, F16.183, F16.188, F16.19, F16.2, F16.20, F16.22, F16.220, F16.221, F16.229, F16.24, F16.240, F16.25, F16.250, F16.251, F16.259, F16.28, F16.280, F16.283, F16.288, F16.29, F18.1, F18.10, F18.12, F18.120, F18.121, F18.129, F18.14, F18.15, F18.150, F18.151, F18.159, F18.17, F18.170, F18.18, F18.180, F18.188, F18.19, F18.2, F18.20, F18.22, F18.220, F18.221, F18.229, F18.24, F18.240, F18.25, F18.250, F18.251, F18.259, F18.27, F18.28, F18.280, F18.288, F18.29, F19.1, F19.10, F19.12, F19.120, F19.121, F19.122, F19.129, F19.14, F19.15, F19.150, F19.151, F19.159, F19.16, F19.160, F19.17, F19.170, F19.18, F19.180, F19.181, F19.182, F19.188, F19.19, F19.2, F19.20, F19.22, F19.220, F19.221, F19.222, F19.229, F19.23, F19.230, F19.231, F19.232, F19.239, F19.24, F19.240, F19.25, F19.250, F19.251, F19.259, F19.26, F19.27, F19.28, F19.280, F19.281, F19.282, F19.288, F19.29
**Opioid poisoning**<br>T400X1, T400X2, T400X3, T400X4, T401X1, T401X2, T401X3, T401X4, T402X1, T402X2, T402X3, T402X4, T403X1, T403X2, T403X3, T403X4, T404X1, T404X2, T404X3, T404X4, T40411, T40412, T40413, T40414, T40421, T40422, T40423, T40424, T40491, T40492, T40493, T40494, T40601, T40602, T40603, T40604, T40691, T40692, T40693, T40694
**Alcohol poisoning**<br>T510
**Cocaine poisoning**<br>T405X1, T405X2, T405X3, T405X4
**Cannabis and derivatives poisoning**<br>T407
**Hallucinogen poisoning**<br>T408, T409
**Benzodiazepine, sedative, hypnotic poisoning**<br>T42
**Psychostimulant poisoning**<br>T436

© 2023 Treitler P et al. *JAMA Health Forum.*

eReference

[1] Knight DK, Blue TR, Flynn PM, Knight K. The TCU Drug Screen 5: Identifying Justice-involved Individuals with Substance Use Disorders. J Offender Rehabil. 2018;57(8):525-537. doi:10.1080/10509674.2018.1549180

© 2023 Treitler P et al. *JAMA Health Forum.*
