# Supplementary material for: Hospital Use and Mortality Among Decarcerated Individuals With Substance Use Disorder After a Large-scale COVID-19 Emergency Prison Release Program
Source: JAMA Health Forum. 2023 Jun 2;4(6):e231200. doi: 10.1001/jamahealthforum.2023.1200 (PMC10238949; doi:10.1001/jamahealthforum.2023.1200)
Supplement: Supplement 2. — Data Sharing Statement [file jamahealthforum-e231200-s002.pdf]

Data Sharing Statement

Treitler. Hospital Use and Mortality Among Decarcerated Individuals With Substance Use Disorder After a Large-Scale COVID-19 Emergency Prison Release Program. *JAMA Health Forum*. Published June 02, 2023. doi:10.1001/jamahealthforum.2023.1200

Data
Data available: No

Additional Information
Explanation for why data not available: The data that support the findings of this study are available from the New Jersey Department of Health, the New Jersey Department of Corrections, and University Correctional Health Care. Restrictions apply to the availability of these data, which were used under data use agreements for this study. SAS code used in analyses is available on request.
